# Metabolic Alterations in NADSYN1-Deficient Cells

**DOI:** 10.3390/metabo13121196

**Published:** 2023-12-12

**Authors:** Nils W. F. Meijer, Johan Gerrits, Susan Zwakenberg, Fried J. T. Zwartkruis, Nanda M. Verhoeven-Duif, Judith J. M. Jans

**Affiliations:** 1 Department of Genetics, Section Metabolic Diagnostics, University Medical Center Utrecht, Lundlaan 6, 3584 EA Utrecht, The Netherlands; n.w.f.meijer@umcutrecht.nl (N.W.F.M.);; 2 Center for Molecular Medicine, University Medical Center Utrecht, Universiteitsweg 100, 3584 CG Utrecht, The Netherlands

**Keywords:** NADSYN1, de novo NAD+ synthesis pathway, NAD+ salvage pathway, Preiss–Handler pathway, NAD+ deficiency, glycolysis, pentose phosphate pathway, polyol pathway, metabolomics

## Abstract

NAD synthetase 1 (encoded by the gene NADSYN1) is a cytosolic enzyme that catalyzes the final step in the biosynthesis of nicotinamide adenine dinucleotide (NAD+) from tryptophan and nicotinic acid. NADSYN1 deficiency has recently been added to the spectrum of congenital NAD+ deficiency disorders. To gain insight into the metabolic consequences of NADSYN1 deficiency, the encoding gene was disrupted in A549 and HEK293T cells, and the metabolome was profiled in the presence of different NAD+ precursors, including tryptophan, nicotinamide and nicotinic acid. We demonstrate that when precursors of the NAD+ salvage pathway in the form of nicotinamide become limiting, NADSYN1 deficiency results in a decline in intracellular NAD+ levels even in the presence of other potential NAD+ sources such as tryptophan and nicotinic acid. As a consequence, alterations in 122 and 69 metabolites are observed in NADSYN1-deficient A549 and HEK293T cells compared to the wild-type cell line (FC > 2 and *p* < 0.05). We thus show that NADSYN1 deficiency results in a metabolic phenotype characterized by alterations in glycolysis, the TCA cycle, the pentose phosphate pathway, and the polyol pathway.

## 1. Introduction

Nicotinamide adenine dinucleotide (NAD+) is an important cellular cofactor that plays a vital role in energy metabolism, enabling glycolysis, and is pivotal for the transfer of electrons in the electron transport chain as part of oxidative phosphorylation. Therefore, regulation of the NAD+ pool is a vital underpinning of normal cellular functioning. Mammalian cells lack the machinery to transfer NAD+ over the plasma membrane and thus depend on its synthesis to maintain the intracellular NAD+ pool. In mammalian cells, NAD+ is synthesized either de novo from tryptophan via the kynurenine pathway, from nicotinic acid (NA) via the Preiss–Handler pathway, from nicotinamide riboside (NR) via the nicotinamide ribose kinase pathway, or from nicotinamide (NAM) via the salvage pathway. Postnatally, de novo NAD+ synthesis from tryptophan occurs mainly in the liver and, to a lesser extent, in the kidneys, which express all enzymes required for the de novo synthesis of NAD+ from tryptophan. Other tissues rely on liver or diet-derived NAM (a product of NAD+ metabolism) as a precursor for NAD+ biosynthesis via the NAD+ salvage pathway [1].

Disorders of NAD+ metabolism are rare inherited metabolic diseases. Currently reported disorders of NAD+ metabolism include 3-hydroxyanthranilate 3,4-dioxygenase (HAAO) deficiency (OMIM #604521) [2,3], kynureninase (KYNU) deficiency (OMIM #605197) [2,3], NAD synthetase 1 (NADSYN1) deficiency (OMIM #608285) [4,5,6,7,8], and nicotinamide mononucleotide adenylyltransferase 1 (NMNAT1) deficiency (OMIM #608700) [9,10,11,12,13]. These disorders are clinically heterogeneous and are characterized, among others, by vertebral, renal, and cardiac defects and blindness.

Recently, various studies reported patients carrying biallelic NADSYN1 variants. NADSYN1 is a cytosolic enzyme that catalyzes the conversion of nicotinic acid adenine dinucleotide (NAAD+) to NAD+. Thus far, 19 patients have been reported as having an inherited defect in NADSYN1, of which 5 five presented with perinatal lethality [4,5,6,7,8]. NADSYN1 is particularly important during embryonic development, as evidenced by defective organogenesis in patients. It is conceivable that the clinical severity of the disease is dependent on the availability of NAD+ precursors, as has been shown for deficiencies of KYNU and HAAO in mice [2]. Despite the central role of NADSYN1 in NAD+ metabolism, NAD+ synthesis, and/or NAD+ salvage, patients with a deficiency have a fairly normal lifespan, indicating that NADSYN1 is largely dispensable postnatally [5,7,8].

Previously, methods to characterize the NAD+ metabolome have been described that revealed the intricate balance between precursor supplementation and NAD+ levels [14,15]. To gain more in-depth insight into the biochemical consequences of NADSYN1 deficiency, we therefore examined the metabolome of NADSYN1-deficient A549 and HEK293T cells using untargeted metabolomics and targeted isotope tracing in the presence of different NAD+ precursors. We show that the genetic deletion of NADSYN1 leads to a reduction in NAD+ under conditions where precursors of the salvage pathway become limiting. Furthermore, we demonstrate that reduced levels of NAD+ as a consequence of NADSYN1 deficiency result in a disturbed glycolysis and subsequent alterations in the TCA cycle, pentose phosphate pathway, and polyol pathway.

## 2. Materials and Methods

### 2.1. Generation of NADSYN1-Deficient HEK293T and A549 Cell Lines

NADSYN1 knockout HEK293T and A549 cells were generated using CRISPR/CAS9. To this end, cells were transiently transfected with pSpCas9(BB)-2A-GFP (PX458) with X-tremegene (Sigma Aldrich, Burlington, VT, USA) according to the manufacturer’s guidelines [16]. All sgRNA sequences used for genome editing of HEK293T and A549 cells were generated using CRISPOR and can be found in Table S1 [17]. Next, GFP-positive cells were sorted using a FaCSaria II flow cytometer (BD). Sorted cells were plated in 10 cm culture dishes at low density. Next, single colonies were picked and screened for genomic alteration of the NADSYN1 locus by sequencing (Figure S1). To this end, genomic DNA was isolated using a QIAamp DNA Micro Kit (QIAGEN). Hereafter, a PCR was performed with the primers displayed in Table S2. The PCR product was isolated from agarose gels using the QIAquick Gel Extraction Kit (QIAGEN) and sent for sequencing (Macrogen, Amsterdam, The Netherlands). 

### 2.2. Cell Lines

HEK293T were cultured in DMEM high glucose GlutaMAX, pyruvate (Gibco) supplemented with 10% heat-inactivated FBS (Thermo Fisher Scientific, Waltham, MA, USA), and 1% P/S antibiotics (Thermo Fisher Scientific). A549 cells were maintained in RPMI 1640 (Gibco) supplemented with 10% heat-inactivated FBS (Thermo Fisher Scientific, Waltham, MA, USA), 2 mM glutamine (Merck) and 1% P/S antibiotics (Thermo Fisher Scientific) in 5% CO_2_ atmosphere at 37 °C. 

### 2.3. Intracellular NAD+ Depletion and Supplementation of (Labeled and Unlabeled) NAD+ Precursors 

NAD+ depletion was achieved by passaging the cells in custom-made RPMI medium lacking tryptophan and nicotinamide (Cell Culture Technologies, Gravesano, Switzerland) supplemented with 10% dialyzed FBS (Thermo Fisher Scientific, Waltham, MA, USA) and 1% P/S antibiotics. Upon reaching confluency cells were seeded in 6 wells plates in the same medium supplied with either nicotinamide 8.4 µM (Sigma-Aldrich) or nicotinic acid 0.5 mM (Sigma-Aldrich) or tryptophan 0.5 mM (Sigma-Aldrich) or ^2H4^nicotinic acid 0.5 mM (CDN Isotopes) or ^2H5^tryptophan 0.5 mM (Cambridge Isotope Laboratories, Andover, MA, USA) and cultured for 48 h.

### 2.4. Metabolite Extraction for Metabolomics

Metabolite extraction was performed by washing the cells twice with PBS (4 °C), followed by incubating the cells in 300 μL of methanol/water (80/20, −80 °C) on dry ice for 20 min. Next, cells were scraped twice (once in an initial 300 μL after which the remaining cells were scraped in an additional 200 μL of methanol/water (80/20, −80 °C), after which the methanol sample was collected into a 1.5 mL Eppendorf tube. The samples were centrifuged (16,200× *g*, 10 min, 4 °C), whereafter, the supernatant was transferred to a new Eppendorf tube. The cell extracts were stored at −80 °C until further use.

### 2.5. LC-MS/MS Analysis of the Kynurenine Pathway

Calibration standards were prepared in the following concentration ranges: n-formylkynurenine (Toronto Research Chemicals, Toronto, ON, Canada), l-kynurenine (Sigma-Aldrich), 3-hydroxykynurenine (Sigma-Aldrich), kynurenic acid (Sigma-Aldrich), 3-hydroxyanthranilic acid (Sigma-Aldrich), anthranilic acid (Sigma-Aldrich), quinolinic acid MedChemExpress), NA (Sigma-Aldrich) and NAAD+ (Sigma-Aldrich) 20 nM to 10 µM and tryptophan (Sigma-Aldrich) and NAD+ (Sigma-Aldrich) 0.2 µM to 100 µM. To this end, 10 μL of the internal standard mixture was added to 50 μL calibration standard or 500 μL of sample (methanol extract). The internal standard working solution consisted of ^2H5^tryptophan (Cambridge Isotope Laboratories) (25 µM) and ^2H4^NAD+ (Toronto Research Chemicals) (50 µM). For isotope tracing analysis, 5-hydroxytryptophan (Sigma-Aldrich) (25 µM) was used as an internal standard instead. Next, samples and calibration standards were evaporated under a flow of nitrogen at 40 °C and reconstituted in 50 μL ultrapure water. Sample analysis was performed using an Ultimate 3000 UHPLC system (Thermo Fisher Scientific) coupled to a Q Exactive™ HF hybrid quadrupole Orbitrap mass spectrometer (Thermo Fisher Scientific). Chromatic separation was performed using a Sunshell RP-Aqua column (150 mm × 3 mm i.d., 2.6 μm; ChromaNik Technologies Inc., Osaka, Japan) at 40 °C. Solvent A consisted of 0.1% formic acid in water, and solvent B of 0.1% formic acid in acetonitrile. The flow rate was 0.6 mL/min with the following elution gradient: isocratic 100% A from 0 to 0.5 min, linear from 100 to 85% A from 0.5 to 2 min, linear from 85 to 75% A from 2 to 2.75 min, linear from 75 to 30% A from 2.75 to 3.5 min, isocratic 30% A from 3.5 to 7 min, linear from 30 to 100% A from 7 to 7.2 min and isocratic 100% A (initial solvent conditions) from 7.2 to 12 to equilibrate the column. Metabolites were detected using an electrospray ionization source operating in both negative and positive ion mode over a scan range of 70–700 mass to charge ratio (*m*/*z*). Scan parameters included resolution of 120.000, AGC target value of 1 × 10^6^, maximum injection time of 200 ms, capillary (kV) 4.0 in both negative and positive ion mode, capillary temperature of 300 °C, and a factor of 50 was used for the S-lens RF level. Xcalibur software (version 3.0; Thermo Fisher Scientific) was used for data acquisition. Peak integration was performed using TraceFinder 4.1 software (Thermo Fisher Scientific).

### 2.6. Untargeted Metabolomics by DI-HRMS

Sample cell extracts (70 µL) were added to 70 µL of internal standard working solution and 60 µL of 0.3% formic acid (Emsure, Darmstadt, Germany) as described in Haijes et al. [18]. Next, samples were filtered using a 96-well filter plate (Pall Corporation, Ann Arbor, USA) preconditioned with methanol and a vacuum manifold. The filtered sample was collected in an Armadillo high-performance 96-well PCR plate (Thermo Fisher Scientific). Untargeted metabolomics was performed using direct infusion high-resolution mass spectrometry (DI-HRMS). To this end, a TriVersa NanoMate system (Advion) operated using Chipsoft software (version 8.3.3, Advion) and coupled to a Q Exactive™ Plus hybrid quadrupole-Orbitrap mass spectrometer (Thermo Fisher Scientific) was used as described previously [18]. Briefly, the automatic aspiration and delivery of samples (13 µL) was achieved by engaging the ESI-Chip with the pipette tip. The nitrogen gas pressure was 0.5 psi, and the spray voltage was 1.6 kV. The total run time was 3 min during which the samples were measured in both negative and positive ion modus over a scan range of 70 to 600 mass to charge ratio (*m*/*z*). Scan parameters included a resolution of 140.000, automatic gain control (AGC) target value of 3 e6, maximum injection time of 200 ms, capillary temperature of 275 °C, S-Lens RF factor of 70, and a sample tray temperature of 18 °C. Data acquisition was performed using Xcalibur software (version 3.0, Thermo Fisher Scientific). Thermo RAW files were converted to mzXML format using ThermoRawFileParser (version 1.1.11, Thermo Fisher Scientific). Next, mzXML files were processed using our customized untargeted metabolomics pipeline (Source code available at https://github.com/UMCUGenetics/DIMS, accessed on 5 July 2023). 

### 2.7. LC-MS/MS Analysis of the Glycolysis and Pentose Phosphate Pathway

The standard calibration curve ranged from 0.2 to 100 μM (intermediates of glycolysis and pentose phosphate pathway) and from 0.1 to 50 μM (NAD+ metabolites). To each calibration standard (50 μL) or sample (500 μL), 20 μL of the internal standard working solution was added. The internal standard working solution contained ^2H3^lactate (100 μM), ^13C3−15N1^serine (100 μM), ^13C6^glucose (133 μM), ^2H3^sodiumpyruvate (255 μM) (Sigma-Aldrich) and ^2h4^NAD+ (25 μM) (Toronto Research Chemicals). Prior to analysis, the samples were evaporated under a flow of nitrogen at 40 °C and reconstituted in 50 μL of ultrapure water/acetonitrile (50%/50%) (Merck). The LC-MS method was performed using an Ultimate 3000 UHPLC system (Thermo Fisher Scientific) in conjunction with a Q Exactive™ HF hybrid quadrupole Orbitrap mass spectrometer (Thermo Fisher Scientific). Chromatic separation was performed using an Atlantis Premier BEH Z-HILIC Column (1.7 µm, 2.1 mm × 100 mm, Waters). Solvent A was 20 mM ammonium bicarbonate (Sigma-Aldrich) in ultrapure H_2_O (pH 9), and solvent B was 20 mM ammonium bicarbonate in acetonitrile. The column temperature was 40 °C with the following flow rate and gradient: linear from 90 to 65% B from 0 to 5 min (flow rate: 0.5 mL/min), isocratic 65% B from 5 to 6 min (flow rate: 0.5 mL/min), linear from 65 to 90% B from 6 to 6.5 min (flow rate: 0.65 mL/min), isocratic 90% B from 6.5 to 12 min (flow rate 0.65 mL/min), and isocratic 90% B from 12 to 12.5 min (flow rate: 0.5 mL/min) to reach starting conditions. Scan parameters included resolution of 120.000, AGC target value of 1 × 10^6^, maximum injection time of 200 ms, capillary (kV) 4 in both positive and negative ion mode, capillary temperature of 350 °C, and a factor of 75 was used for the S-lens RF level. Data were acquired using Xcalibur software (version 3.0; Thermo Fisher Scientific). For the integration of raw data peaks, TraceFinder 4.1 software (Thermo Fisher Scientific) was used.

### 2.8. GC-FID Analysis of Sugar Alcohols

Gas-chromatography–flame ionization detection (GC-FID) was performed to measure sorbitol. The standard calibration curve ranged from 0 µM to 100 µM. To each calibration standard (500 μL) and sample (500 μL), 100 μL internal standard solution (3.9 mM ß-d-phenylglucoside, Sigma-Aldrich) was added. Hereafter, calibration standards and methanol extracts were evaporated under a flow of nitrogen at 40 °C and reconstituted in a solution of Tri-Sil TBT (Trimethylsilyl N-trimethylsilylacetamidate, 1-(Trimethylsilyl)-1 H-imidazole, Chlorotrimethylsilane) (Supel-co) and ultrapure water (300 μL). To derivatize the samples, samples were maintained at 105 °C for 30 min. Next, 500 μL hexane (VWR) and 1 mL ultrapure water were added, after which the samples were vortexed and centrifuged for 5 min at 3000 rpm. The supernatant was then transferred to a new tube, mixed with 1 mL 0.1 M HCl, and centrifuged at 3000 rpm for 5 min. Next, the supernatant was transferred to a new tube, after which Anhydrous Sodium Sulfate (Sigma-Aldrich) and subsequently N,O-Bis(Trimethylsilyl)Trifluoroacetamide (Sigma-Aldrich) were added. The GC-MS method was performed using a Trace 1300 GC-FID (Thermo Fisher Scientific) in conjunction with an iConnect flame ionization detector (FID). Samples (2 µL) were automatically injected using an AI/AS 3000 autosampler and split/spitless injector system. During sample injection, the split mode was 1:15 with a temperature of 280 °C. Separation was performed using an HP-1 column (25 m × 0.2 mm; 0.11 µm; Agilent Technologies, J&W Scientific) coupled to the FID at a temperature of 300 °C. The carrier gas was helium, with a flow rate of 1 mL/min. The oven temperature conditions were as follows: 120 °C for 10 min, increased to 265 °C at 3 °C/min, increased to 295 °C at 10 °C/min, and 295 °C for 5 min. For the integration of raw data peaks, Empower software 3.6.0 (Waters) and Chromeleon software 7.2.10 (Thermo Fisher Scientific) were used.

### 2.9. Statistical Analysis

Statistical analysis was performed using GraphPad Prism 9.3.0 and MetaboAnalyst 5.0. The parametric unpaired, two-tailed *t*-test was used in GraphPad Prism to compare the means of two samples. Principal component analysis (PCA) and heat map analysis (*t*-test based) were conducted in MetaboAnalyst. All cellular experiments were conducted using three independent replicates, of which the values are shown as the mean ± standard deviation. A *p* value of <0.05 was considered statistically significant.

## 3. Results

### 3.1. Nicotinamide and Nicotinic Acid but Not Tryptophan Are a Source of NAD+ in HEK293T and A549 Cells

**Figure 1:**
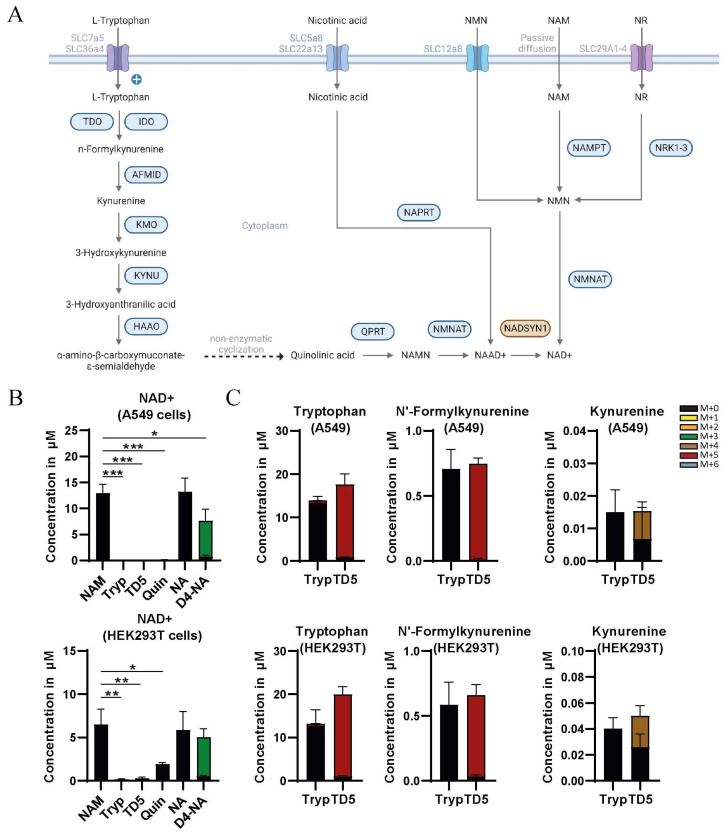
(**A**) Graphical depiction of NAD+ synthesis, including the de novo synthesis pathway, the Preiss–Handler pathway, the nicotinamide ribose kinase pathway, and the salvage pathway. Adapted from “Overview of BCAA Catabolic Enzymes” by BioRender.com (2023). Retrieved from https://app.biorender.com/biorender-templates (accessed on 24 November 2023). (**B**) Total (labeled and unlabeled) concentration of NAD+ in A549 and HEK293T cells cultured in the presence of either 8.4 µM nicotinamide (NAM) or 0.5 mM tryptophan (Tryp) or 0.5 mM ^2H5^tryptophan (TD5) or 0.5 mM quinolinic acid (Quin) or 0.5 mM nicotinic acid (NA) or 0.5 mM ^2H4^nicotinic acid (D4-NA) (mean ± SD, *n* = 3, *t*-test with *, *p* < 0.05; **, *p* < 0.01; ***, *p* < 0.001; and ****, *p* < 0.0001). (**C**) Total (labeled and unlabeled) concentration of tryptophan, n’-formylkynurenine, and kynurenine after 48 h incubation with 0.5 mM tryptophan (Tryp) or 0.5 mM ^2H5^tryptophan (TD5) in A549 and HEK293T cells (mean ± SD, *n* = 3). Isotopologues are described as follows: unlabeled (M + 0), containing 1 deuterium ^2^H atom (M + 1), etc.

To assess the biochemical consequences of NADSYN1 deficiency, we generated A549 and HEK293T cells lacking NADSYN1 and examined components of the NAD+ metabolism (Figure 1A). The expression of enzymes in NAD+ metabolism varies between cell types [19,20,21]. To identify which sources contribute to NAD+ levels in HEK293T and A549 cells, we determined the concentration of NAD+-related metabolites in wild-type and NADSYN1-deficient cells cultured in precursor-free medium supplemented with tryptophan, ^2H5^tryptophan, quinolinic acid, nicotinic acid (NA), or ^2H4^nicotinic acid. Nicotinamide (NAM) is a major source of NAD+ in cultured cells and was therefore used as a control [1,19]. Incubation with either NA or NAM for 48 h led to an increase in the intracellular NAD+ concentration, consistent with the expression of NAPRT and NAMPT in these cells (Figure 1B) [20,21,22]. Replacing NA for ^2H4^NA led to a similar increase in NAD+ levels and resulted in 95% labeling of the total NAD+ pool in A549 and HEK293T cells (Figure 1B). In line with previous observations, 95% of labeled NAD+ was M + 3 due to loss of the redox-active hydrogen at position 4 [1]. The addition of quinolinic acid resulted in a lower level of NAD+ only in HEK293T cells (Figure 1B). In contrast, incubation with tryptophan or ^2H5^tryptophan did not affect the NAD+ level nor the labeled fraction of NAD+ in A549 and HEK293T cells (Figure 1B), suggesting tryptophan is not a source for NAD+ in these cells. Label incorporation from ^2H5^tryptophan was observed in only two metabolites, n’-formylkynurenine, and kynurenine, even though the tryptophan pool consisted almost entirely of M + 5 tryptophan (Figure 1C). This indicates a block downstream of kynurenine in both cell lines. Overall, we conclude that A549 and HEK293T cells cannot synthesize NAD+ from tryptophan.

### 3.2. Precursor Availability Determines Impact of NADSYN1 Deficiency

**Figure 2:**
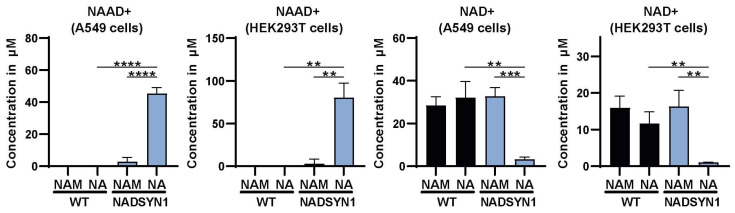
Total concentration of NAAD+ and NAD+ in wild-type and NADSYN1-deficient A549 and HEK293T cells cultured in the presence of either 8.4 µM nicotinamide (NAM) or 0.5 mM nicotinic acid (NA) for 48 h (mean ± SD, *n* = 3, *t*-test with *, *p* < 0.05; **, *p* < 0.01; ***, *p* < 0.001; and ****, *p* < 0.0001).

To determine the impact of NADSYN1 deficiency on cellular metabolism, we first established under which conditions cells become reliant on NADSYN1 for maintaining the intracellular NAD+ pool. To this end, we examined NAD+ levels by LC-MS/MS in wild-type and NADSYN1-deficient HEK293T and A549 cells cultured in the presence of different NAD+ sources (nicotinic acid and nicotinamide). Tryptophan was excluded as it is not used as a source for NAD+ in these cell lines. Since the conversion of nicotinic acid to NAD+ is NADSYN1-dependent, we expected a decline in NAD+ in NADSYN1-deficient cells exposed to NA as the only NAD+ precursor. Indeed, cellular NAD+ levels in NADSYN1-deficient A549 and HEK293T cells declined by 89% and 93% compared to wild-type cells, whereas NAAD+, the substrate of NADSYN1, accumulated (Figure 2). Thus, NADSYN1-deficient cells develop NAD+ deficiency under conditions in which precursors for the salvage pathway are not available.

### 3.3. Reduced NAD+ Levels as a Consequence of NADSYN1 Deficiency Alter Flux through Glycolysis and Branching Pathways

**Figure 3:**
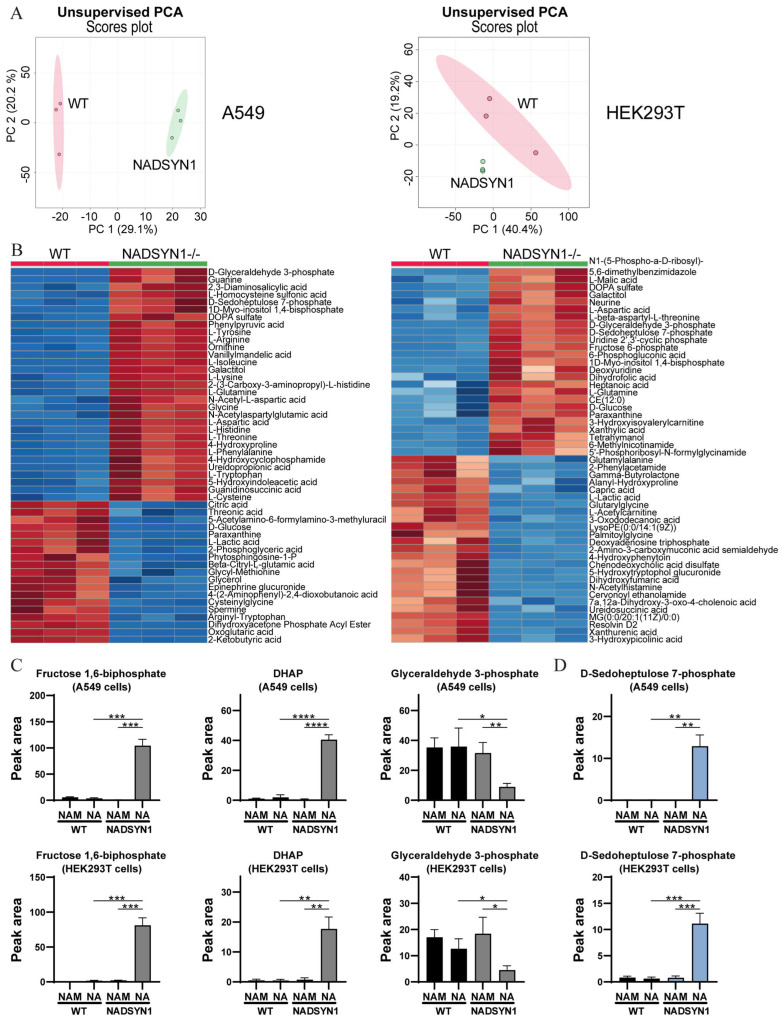
(**A**) Unsupervised principal component analysis of wild-type and NADSYN1-deficient A549 and HEK293T cells cultured in the presence of 0.5 mM nicotinic acid (*n* = 3). (**B**) Heatmap analysis (based on *t*-test *p* < 0.05) of most significantly changed metabolites in wild type and NADSYN1-deficient A549 and HEK293T cells cultured in the presence of 0.5 mM nicotinic acid. Blue areas represent a reduced intensity of the respective metabolite, while red areas represent an increased intensity (*n* = 3). (**C**,**D**) Total concentration of fructose 1,6-biphosphate, DHAP, glyceraldehyde 3-phosphate (C), and sedoheptulose 7-phosphate (**D**) in wild-type and NADSYN1-deficient A549 and HEK293T cells cultured in the presence of 8.4 µM nicotinamide (NAM) or 0.5 mM nicotinic acid (NA) for 48 h (mean ± SD, *n* = 3, *t*-test with *, *p* < 0.05; **, *p* < 0.01; ***, *p* < 0.001; and ****, *p* < 0.0001).

In view of the central role of NAD(P)+ cofactors in modulating a plethora of cellular processes, the observed decline in NAD+ levels in NADSYN1-deficient cells is expected to have a major effect on a variety of metabolic processes. In order to explore how NADSYN1 deficiency globally alters cellular metabolism, we analyzed the metabolomes of HEK293T and A549 WT and NADSYN1−/− cells cultured in the presence of nicotinic acid in medium without NAM with direct infusion high-resolution mass spectrometry. We detected 1913 unique mass peaks corresponding to 3929 metabolites. To determine whether NADSYN1-dependent NAD+ deficiency causes a distinct metabolic profile, we performed an unsupervised principal component analysis with MetaboAnalyst (Figure 3A) [23]. As expected, NADSYN1-deficient cells clustered distinctly from wild-type cells for both A549 and HEK293T cells. To explore what metabolic pathways are most strongly affected by NADSYN1-dependent NAD+ deficiency, we identified the most significantly differing metabolites by heat map analysis based on the *t*-test. As shown in Figure 3B, metabolites involved in glycolysis and TCA cycle were significantly altered, confirming NADSYN1 deficiency impacts cellular energy metabolism under conditions in which salvage pathway dependent NAD+ sources become limited. Here, the decline in NAD+ levels is expected to diminish the conversion of glyceraldehyde 3-phosphate (GA3P) to 1,3-biphosphoglycerate by GAPDH. To assess the impact of NADSYN1-dependent NAD+ deficiency on glycolysis and side branches of the glycolytic pathway, we examined intermediates of the pathway by targeted metabolic profiling. In line with the dependency of GAPDH for NAD+, we observed an increase in metabolites preceding GAPDH, including fructose 6-P, fructose 1,6-BP and DHAP (Figure 3C). Interestingly, GA3P, the substrate of GAPDH, was reduced in NAD+-deficient cells, which may indicate an increased flux toward Glycerol 3-phosphate dehydrogenase (GPD1) to promote regeneration of NAD+. 

The pentose phosphate pathway is a metabolic route diverting from glycolysis at the level of glucose 6-P or fructose 6-P and glyceraldehyde 3-phosphate. It generates pentoses and NADPH, which are important for the formation of fatty acids, nucleotides, and sterols. Given the accumulation of intermediates in the first steps of glycolysis in NAD+-deficient cells, we further examined metabolites of the PPP. As shown in Figure 3, the genetic deletion of NADSYN1 led to a significant increase in the levels of sedoheptulose 7-P compared to the WT cell lines. The addition of nicotinamide abolished the effect, indicating that NAD+ deficiency underlies disturbed glycolysis in NADSYN1-deficient cells and results in a carbon overflow toward the pentose phosphate pathway.

### 3.4. Reduced NAD+ Levels as a Consequence of NADSYN1 Deficiency Result in Accumulation of Sorbitol

**Figure 4:**
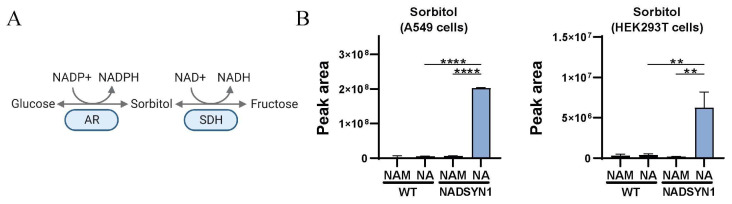
(**A**) Graphical representation of the polyol pathway. Adapted from “Overview of BCAA Catabolic Enzymes” by BioRender.com (2023). Retrieved from https://app.biorender.com/biorender-templates (accessed on 1 December 2023). (**B**) Total peak area of sorbitol in wild-type and NADSYN1-deficient A549 and HEK293T cells cultured in the presence of 8.4 µM nicotinamide (NAM) or 0.5 mM nicotinic acid (NA) for 48 h (mean ± SD, *n* = 3, *t*-test with *, *p* < 0.05; **, *p* < 0.01; ***, *p* < 0.001; and ****, *p* < 0.0001).

Next to the PPP, alterations in the polyol pathway are expected when glycolysis is impaired as a consequence of lowered NAD+ levels. The polyol pathway consists of a two-step process in which glucose is converted to sorbitol in a reaction catalyzed by the enzyme aldolase reductase. Aldose reductase has a low affinity for glucose. Therefore, under normal glucose levels, the activity of aldose reductase and thus the conversion of glucose to sorbitol is relatively low. The second step of the pathway is the transformation of sorbitol to fructose, which is catalyzed by sorbitol dehydrogenase in an NAD+-dependent manner. Untargeted metabolic analysis of A549 and HEK293T NADSYN1−/− cells in precursor-free medium supplemented with nicotinic acid displayed an increase in a compound whose mass corresponded to sorbitol. Using GC-FID, we validated that, indeed, sorbitol is accumulating in these cells (Figure 4).

## 4. Discussion

NADSYN1 deficiency is an inherited metabolic disorder that results in a deficiency of NAD+ during embryogenesis [4]. Here, we examined the cellular pathophysiology of NADSYN1 deficiency using A549 and HEK293T cells, two cell types that are frequently used in metabolic studies. We show that NA and NAM are a source of NAD+ in HEK293T and A549 cells while tryptophan cannot be used as a precursor to supply NAD+. Previously, it has been established that mouse embryos deficient in HAAO or KYNU develop congenital malformations only when limitations are placed on their diet with respect to NA and NAM [2]. This suggests that in the presence of sufficient NAD+ precursors, the production of cellular NAD+ via alternative pathways can compensate for the lack of de novo synthesis, even prenatally. It is therefore conceivable that NADSYN1 deficiency, in a similar manner, predisposes the individual to NAD+ deficiency, while the eventual outcome depends on the availability of alternative precursors such as NAM and NR. Indeed, nicotinamide is used as a treatment for congenital NAD+ deficiencies and has been used to increase plasma NAD+ levels in a patient suffering from NADSYN1 deficiency [8]. 

Our findings are in agreement with those of Xiao et al. that most immortalized cell lines do not utilize the de novo synthesis pathway to generate NAD+ [19]. This may also reflect the situation in most postnatal tissues, including the ones most severely affected by NADSYN1 deficiency. Whether de novo synthesis is functional and important to maintain NAD+ in the cellular progenitors of affected tissues during embryonic development or that NAD+ deficiency occurs as part of a non-autonomous process by which NAM (as a product of tryptophan-derived NAD+ metabolism) is released to a lesser extent by other cells is unknown and requires further investigation. 

While the discovery that NADSYN1 deficiency severely affects development in humans highlights the importance of NADSYN1 and its role in maintaining cellular NAD+ levels, little is known about the NAD+-dependent processes responsible for defective embryonic development [4,5,6,7,8]. Our data reveal that glycolysis is significantly inhibited at the level of GAPDH, resulting in an accumulation of upstream glycolytic intermediates. Interestingly, the level of its substrate, glyceraldehyde 3-phosphate, was reduced while DHAP accumulated in NAD+-deficient cells and probably reflects an increased flux toward the glycerol phosphate shuttle. Previously, a study showed that DHAP is a potent inhibitor of ACMS decarboxylase (ACMSD), the activity of which is important for controlling the metabolic fate of tryptophan along the de NAD+ de novo synthesis pathway [24]. While de novo NAD+ synthesis is not functional in these cells, the regulation of ACMSD by DHAP might be a potential mechanism to regulate NAD+ levels that was lost in A549 and HEK293T cells. Although our results point to a disturbed glycolysis and TCA cycle as putative causes for malformations, it is well possible that any of the other NAD+-dependent reactions is more important. 

Consistent with the loss of NADSYN1, NAAD+, the substrate of NADSYN1, accumulated when only NA was supplied as an NAD+ precursor. While the exact contribution of NAAD+ to the pathology of NADSYN1 deficiency remains unknown, NAAD+ could potentially be used as a new diagnostic marker. Consistent with altered glycolysis and accumulation of glycolytic intermediates preceding GAPDH in the absence of NADSYN1, glucose-derived carbons are exceedingly diverted via alternative pathways, including the PPP, resulting in an accumulation of sedoheptulose 7-P. Supplementation of NADSYN1-deficient cells with nicotinamide restored the level of PPP intermediates, suggesting that accumulation of sedoheptulose 7-P is caused by diminished levels of NAD+. Whether the accumulation of PPP intermediates is a secondary consequence of the altered glycolysis or caused by a reduction in the levels of NAPD(H) requires further exploration. Increased levels of sedoheptulose 7-P have been previously reported in various cases of Transaldolase deficient patients [25]. However, little is known about the manner in which sedoheptulose 7-P affects the disease. Further examination revealed that sorbitol accumulated in NADSYN1-deficient cells, indicating a blockade in the polyol pathway at the sorbitol dehydrogenase step. Accumulation of sorbitol seems not to be a major factor impacting embryonic development based on studies in rats [26,27]. Eriksson et al. showed that the accumulation of sorbitol as a consequence of fructose supplementation to rat embryos results in normal development [27]. Consistent with this, patients suffering from sorbitol dehydrogenase deficiency often develop symptoms at later stages in life, including neuropathy and foot deformities [28]. These findings suggest that the intracellular accumulation of sorbitol likely is not a major contributing factor to the congenital malformations observed in NAD+ deficiency but might affect patients suffering from NADSYN1 deficiency later in life.

Overall, utilizing NAD+ tracing methods in combination with untargeted metabolomics, we demonstrate that reduced NAD+ levels in NADSYN1-deficient cells result in a blockade in glycolysis, which leads to an overflow of carbons to the pentose phosphate pathway and polyol pathway. In addition, sufficient availability of NAD+ precursors to the salvage pathway may rescue the metabolic phenotype of NADSYN1 deficiency.

## 5. Limitations of the Study

We acknowledge that our in vitro model has some limitations. The research presented in this study is based on the use of cell lines and may therefore not closely represent primary cells. Additionally, metabolic alterations, as detected by DI-HRMS seen in A549 and HEK293T cells following the loss of NADSYN1, are only partially overlapping (Figure S2). As we only used a single sgRNA in HEK293T cells (in contrast to A549 cells), additional NADSYN1-deficient HEK293T cells should be generated to see if the distinct metabolic alterations reflect different cellular backgrounds. Another possible explanation is the off-target effects of the used sgRNA. However, all metabolic changes discussed in detail here are shared by A549 and HEK293T cells. Most relevant findings have been confirmed by targeted mass spectrometry approaches; DI-HRMS revealed many more changes that require further confirmation. Similarly, changes may have gone unnoticed if not correctly annotated. 

## Data Availability

The datasets presented in this study are available upon request to the corresponding author. Due to specific nature of the data formats and requirement for the correct accompanying code to process it, the raw data underlying the datasets presented in this study are available upon request to the corresponding author.

## Supplementary Materials

The following supporting information can be downloaded at https://www.mdpi.com/article/10.3390/metabo13121196/s1. Table S1: Oligonucleotide sequences used to generate NADSYN1-deficient A549 and HEK293T cells. Figure S1: Sequences derived from genomic DNA of wild type and NADSYN1-deficient A549 and HEK293T cells. Table S2: Primer sequences used to perform PCR on A549 wild type and NADSYN1-deficient A549 and HEK293T cells. Figure S2: Venn diagram depicting the overlap between significantly (based on FC > 2 and *p* < 0.05) altered metabolites between A549 and HEK293T cells as a consequence of NADSYN1−/− dependent NAD+ deficiency. The 19 overlapping metabolites are represented in the table with an additional column indicating whether a metabolite is increased or decreased in A549 and HEK293T cells as a consequence of NADSYN1−/− dependent NAD+ deficiency.

## References

[1] Liu L., Su X., Quinn W.J., Hui S., Krukenberg K., Frederick D.W., Redpath P., Zhan L., Chellappa K., White E. (2018). Quantitative Analysis of NAD Synthesis-Breakdown Fluxes. Cell Metab..

[2] Shi H., Enriquez A., Rapadas M., Martin E.M., Wang R., Moreau J., Lim C.K., Szot J.O., Ip E., Hughes J.N. (2017). NAD Deficiency, Congenital Malformations, and Niacin Supplementation. N. Engl. J. Med..

[3] Szot J.O., Slavotinek A., Chong K., Brandau O., Nezarati M., Cueto-González A.M., Patel M.S., Devine W.P., Rego S., Acyinena A.P. (2021). New cases that expand the genotypic and phenotypic spectrum of Congenital NAD Deficiency Disorder. Hum. Mutat..

[4] Szot J.O., Campagnolo C., Cao Y., Iyer K.R., Cuny H., Drysdale T., Flores-Daboub J.A., Bi W., Westerfield L., Liu P. (2020). Bi-allelic Mutations in NADSYN1 Cause Multiple Organ Defects and Expand the Genotypic Spectrum of Congenital NAD Deficiency Disorders. Am. J. Hum. Genet..

[5] Lin J., Zhao L., Zhao S., Li S., Zhao Z., Chen Z., Zheng Z., Shao J., Niu Y., Li X. (2021). Disruptive NADSYN1 Variants Implicated in Congenital Vertebral Malformations. Genes.

[6] Kortbawi H., Ames E., Pritchard A., Devine P., van Ziffle J., Slavotinek A. (2022). Further description of two patients with biallelic variants in NADSYN1 in association with cardiac and vertebral anomalies. Am. J. Med. Genet. A.

[7] Aubert-Mucca M., Janel C., Porquet-Bordes V., Patat O., Touraine R., Edouard T., Michot C., Tessier A., Cormier-Daire V., Attie-Bitach T. (2023). Clinical heterogeneity of NADSYN1-associated VCRL syndrome. Clin. Genet..

[8] Erbs E., Brasen C.L., Lund A.M., Rasmussen M. (2023). Adult patient diagnosed with NADSYN1 associated congenital NAD deficiency and analysis of NAD levels to be published in: European Journal of Medical Genetics. Eur. J. Med. Genet..

[9] Koenekoop R.K., Wang H., Majewski J., Wang X., Lopez I., Ren H., Chen Y., Li Y., Fishman G.A., Genead M. (2012). Mutations in NMNAT1 cause Leber congenital amaurosis and identify a new disease pathway for retinal degeneration. Nat. Genet..

[10] Khan A.O., Budde B.S., Nürnberg P., Kawalia A., Lenzner S., Bolz H.J. (2018). Genome-wide linkage and sequence analysis challenge CCDC66 as a human retinal dystrophy candidate gene and support a distinct NMNAT1-related fundus phenotype. Clin. Genet..

[11] Nash B.M., Symes R., Goel H., Dinger M.E., Bennetts B., Grigg J.R., Jamieson R.V. (2018). NMNAT1 variants cause cone and cone-rod dystrophy. Eur. J. Hum. Genet..

[12] Bedoni N., Quinodoz M., Pinelli M., Cappuccio G., Torella A., Nigro V., Testa F., Simonelli F., Corton M., Lualdi S. (2020). An Alu-mediated duplication in NMNAT1, involved in NAD biosynthesis, causes a novel syndrome, SHILCA, affecting multiple tissues and organs. Hum. Mol. Genet..

[13] Bedoukian E.C., Zhu X., Serrano L.W.B., Scoles D., Aleman T.S. (2022). Nmnat1-Associated Cone-Rod Dystrophy: Evidence for a Spectrum of Foveal Maldevelopment. Retin. Cases Brief Rep..

[14] Bustamante S., Jayasena T., Richani D., Gilchrist R.B., Wu L.E., Sinclair D.A., Sachdev P.S., Braidy N. (2017). Quantifying the cellular NAD+ metabolome using a tandem liquid chromatography mass spectrometry approach. Metabolomics.

[15] Cuny H., Kristianto E., Hodson M.P., Dunwoodie S.L. (2021). Simultaneous quantification of 26 NAD-related metabolites in plasma, blood, and liver tissue using UHPLC-MS/MS. Anal. Biochem..

[16] Ran F.A., Hsu P.D., Wright J., Agarwala V., Scott D.A., Zhang F. (2013). Genome engineering using the CRISPR-Cas9 system. Nat. Protoc..

[17] Concordet J.P., Haeussler M. (2018). CRISPOR: Intuitive guide selection for CRISPR/Cas9 genome editing experiments and screens. Nucleic Acids Res..

[18] Haijes H.A., Willemsen M., Van der Ham M., Gerrits J., Pras-Raves M.L., Prinsen H.C.M.T., Van Hasselt P.M., De Sain-van der Velden M.G.M., Verhoeven-Duif N.M., Jans J.J.M. (2019). Direct Infusion Based Metabolomics Identifies Metabolic Disease in Patients’ Dried Blood Spots and Plasma. Metabolites.

[19] Xiao Y., Elkins K., Durieux J.K., Lee L., Oeh J., Yang L.X., Liang X., DelNagro C., Tremayne J., Kwong M. (2013). Dependence of tumor cell lines and patient-derived tumors on the NAD salvage pathway renders them sensitive to NAMPT inhibition with GNE-618. Neoplasia.

[20] Hara N., Yamada K., Shibata T., Osago H., Hashimoto T., Tsuchiya M. (2007). Elevation of cellular NAD levels by nicotinic acid and involvement of nicotinic acid phosphoribosyltransferase in human cells. J. Biol. Chem..

[21] Cole J., Guiot M.C., Gravel M., Bernier C., Shore G.C., Roulston A. (2017). Novel NAPRT specific antibody identifies small cell lung cancer and neuronal cancers as promising clinical indications for a NAMPT inhibitor/niacin co-administration strategy. Oncotarget.

[22] Duarte-Pereira S., Pereira-Castro I., Silva S.S., Correia M.G., Neto C., da Costa L.T., Amorim A., Silva R.M. (2016). Extensive regulation of nicotinate phosphoribosyltransferase (NAPRT) expression in human tissues and tumors. Oncotarget.

[23] Pang Z., Chong J., Zhou G., de Lima Morais D.A., Chang L., Barrette M., Gauthier C., Jacques P.-É., Li S., Xia J. (2021). MetaboAnalyst 5.0: Narrowing the gap between raw spectra and functional insights. Nucleic Acids Res..

[24] Garavaglia S., Perozzi S., Galeazzi L., Raffaelli N., Rizzi M. (2009). The crystal structure of human alpha-amino-beta-carboxymuconate-epsilon-semialdehyde decarboxylase in complex with 1,3-dihydroxyacetonephosphate suggests a regulatory link between NAD synthesis and glycolysis. FEBS J..

[25] Huck J.H., Struys E.A., Verhoeven N.M., Jakobs C., van der Knaap M.S. (2003). Profiling of pentose phosphate pathway intermediates in blood spots by tandem mass spectrometry: Application to transaldolase deficiency. Clin. Chem..

[26] Eriksson U.J., Naeser P., Brolin S.E. (1986). Increased accumulation of sorbitol in offspring of manifest diabetic rats. Diabetes.

[27] Eriksson U.J., Brolin S.E., Naeser P. (1989). Influence of sorbitol accumulation on growth and development of embryos cultured in elevated levels of glucose and fructose. Diabetes Res..

[28] Cortese A., Zhu Y., Rebelo A.P., Negri S., Courel S., Abreu L., Bacon C.J., Bai Y., Bis-Brewer D.M., Bugiardini E. (2020). Biallelic mutations in SORD cause a common and potentially treatable hereditary neuropathy with implications for diabetes. Nat. Genet..

