# Supplementary material for: Metabolic Alterations in NADSYN1-Deficient Cells
Source: Metabolites. 2023 Dec 12;13(12):1196. doi: 10.3390/metabo13121196 (PMC10745350; doi:10.3390/metabo13121196)
Supplement: Supplementary file 1 [file metabolites-13-01196-s001.zip › metabolites-2705250-supplementary.pdf]

Supplementary Materials

Table S1. Oligonucleotide sequences used to generate NADSYN1 deficient A549 and HEK293T cells

**NADSYN1 #1**	Forward: GCAAATTGCCCTCGAAGTCC<br>Reverse: GGACTTCGAGGGCAATTTGC
**NADSYN1 #2**	Forward: GCAGGACATCATCTGCGACGT<br>Reverse: ACGTCGCAGATGATGTCCTGC

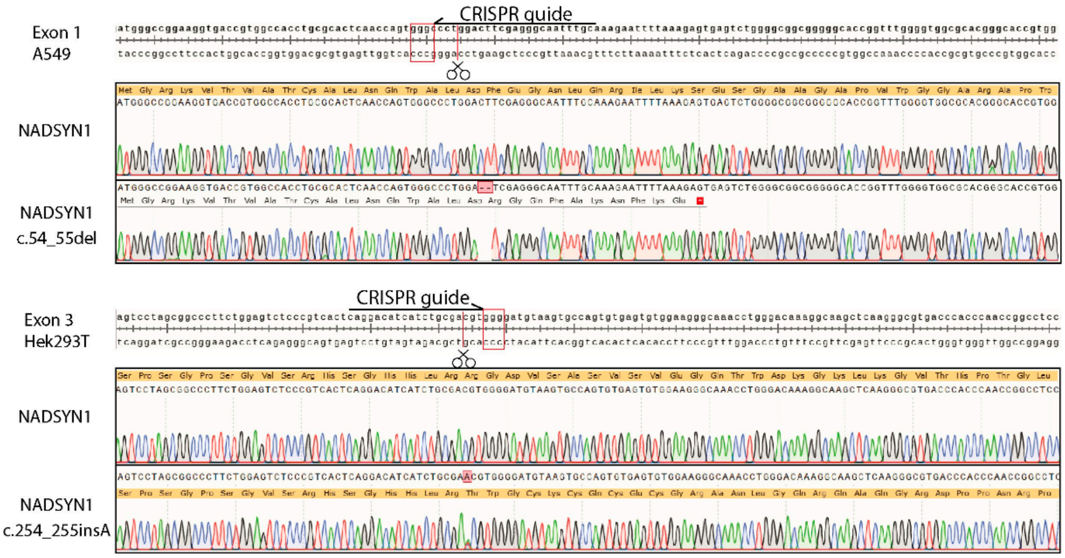

Figure S1. Sequences derived from genomic DNA of wild type and NADSYN1 deficient A549 and HEK293T cells.

Table S2. Primer sequences

**NADSYN1 #1**	Forward: gaccctggtcttgctgtcc<br>Reverse: cctgacctcaggtgacttcc
**NADSYN1 #2**	Forward: gctgctcattggaatcacct<br>Reverse: ttacggggctgagataggaa

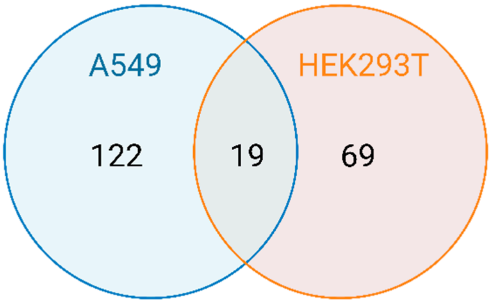

**Metabolite**	**A549**	**HEK293T**
1D-Myo-inositol 1,4-bisphosphate	Increased	Increased
2-Amino-3-carboxymuconic acid semialdehyde	Decreased	Decreased
2-Hexenoylcarnitine	Decreased	Decreased

D-Glyceraldehyde 3-phosphate	Increased	Increased
DOPA sulfate	Increased	Increased
D-Sedoheptulose 7-phosphate	Increased	Increased
Fructose 6-phosphate	Increased	Increased
Galactitol	Increased	Increased
Gluconolactone	Decreased	Decreased
Guanosine	Increased	Increased
L-Homocysteine sulfonic acid	Increased	Increased
Oxoglutaric acid	Decreased	Decreased
Prostaglandin E2	Decreased	Decreased
Sedoheptulose 1,7-bisphosphate	Increased	Increased
Ureidosuccinic acid	Decreased	Decreased
Uridine 2',3'-cyclic phosphate	Increased	Increased
(S)-5-Diphosphomevalonic acid	Decreased	Increased
Deoxyuridine	Decreased	Increased
Inosine	Increased	Decreased

Figure S2. Venn diagram depicting the overlap between significantly (based on FC>2 and P<0.05) altered metabolites between A549 and HEK293T cells as a consequence of NADSYN1-/- dependent NAD+ deficiency. The 19 overlapping metabolites are represented in the table with an additional column indicating whether a metabolite is increased or decreased in A549 and HEK293T cells as a consequence of NADSYN1-/- dependent NAD+ deficiency. Adapted from "Venn Diagram with Organs (Layout)", by BioRender.com (2023). Retrieved from https://app.biorender.com/biorender-templates.
